# Rickettsial Infection in the COVID-19 Era: The Correlation between the Detection of *Rickettsia aeschlimannii* in Ticks and Storytelling Photography of a Presumable Human Rickettsiosis Case

**DOI:** 10.3390/microorganisms11112645

**Published:** 2023-10-27

**Authors:** Donato Antonio Raele, Maria Assunta Cafiero

**Affiliations:** Istituto Zooprofilattico Sperimentale della Puglia e della Basilicata, 71121 Foggia, Italy; mariaassunta.cafiero@izspb.it

**Keywords:** *Rickettsia aeshlimannii*, tick-borne rickettsioses, *tache noire*, *Hyalomma marginatum*

## Abstract

*Rickettsia aeschlimannii* infection is an emerging human tick-borne disease with only a few recorded cases. We reported a presumable autochthonous case of rickettsiosis in an Italian cattle breeder associated with a *Hyalomma marginatum* bite. *Rickettsia aeschlimannii* DNA was detected in both the tick specimen from the patient and the grazing cattle close to his farm.

## 1. Introduction

*Rickettsia aeschlimannii* is an emerging human tick-borne pathogen responsible for typical signs of Mediterranean spotted fever (MSF), acute hepatitis without MSF-like signs, and fever with meningitis without inoculation eschar or cutaneous rash [1,2,3,4]. The species was isolated for the first time in 1997 from infected *Hyalomma marginatum* ticks and characterized as a new member of Spotted Fever Group (SFG) rickettsiae [5]. Subsequently, *R. aeschlimannii* was linked to a human infection case in a French traveler returning from Morocco [6] and it has recently been identified in several species of ticks collected from animals and humans in the temperate climate area of Eurasia and Africa [7,8,9,10]. In Europe, human infections caused by *R. aeschlimannii* are mostly linked to imported cases with only one autochthonous clinical case recorded in Italy [3]. Nevertheless, in Italy, *R. aeschlimannii* DNA has been frequently detected in several species of ticks removed from mammals and birds [11,12]. We report the results of a retrospective detection of a tick-borne pathogen involved in a presumptive autochthonous case of rickettsioses in a male cattle breeder in Italy.

## 2. The Study

**Figure 1:**
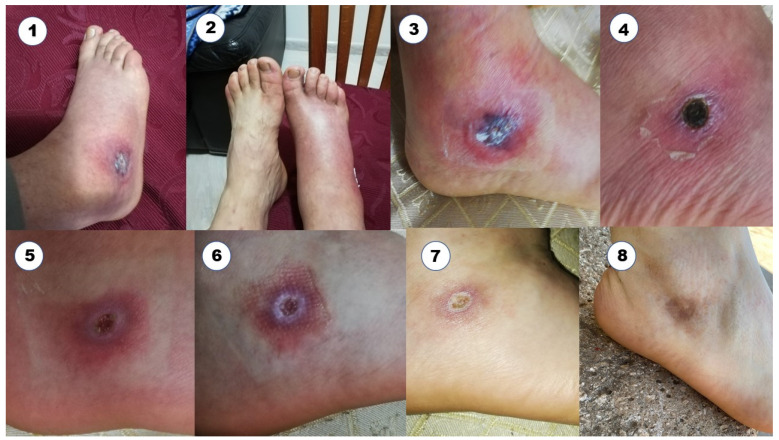
Right foot of the 75-year-old cattle farmer after the tick bite. The sequence of the eight photographs show the evolution of the skin lesions from the 7th day after the tick removal to about two years after: 1 and 2 (4 October 2020—7th); 3 (5 October 2020—8th); 4 (25 October 2020—28th); 5 (12 November 2020—47th); 6 (18 November 2020—53rd); 7 (1 December 2020—66th); 8 (12 May 2022—593rd).

During May 2022, a 75-year-old cattle breeder, male, living in a rural area of Apulia region (Gargano promontory) and without travel history delivered to our laboratory one tick specimen to be analyzed for pathogens. The ectoparasite was stored in 70% ethanol in a plastic vial dated 27 September 2020. The late delivery of the sample was due to the COVID-19 restrictions and fear of the contagion at the time. Questioned, the patient referred that the ectoparasite had been manually removed from his foot during the requested medical consultation; in the occasion, a therapy with antibiotic ointment and amoxicillina 1000 mg daily was also prescribed. On the fourth day, despite the ongoing antibiotic treatment, fever (peak 38.2) and foot pain and swelling accompanied by itch appeared; in the following days, the tick-bite area became progressively darker. The patient did not remember the presence of a maculopapular skin rash associated with the fever but he reported (also to the doctor) the development of an expanding dermal redness at the place of the tick bite. The subsequent therapy with doxycycline 200 mg daily for 14 days gradually led to remission of the referred symptoms. To prove the story, he also provided a series of photos documenting the developed lesions on the tick-bite site in the period October–November 2020; in addition, a more recent photo was also taken at that date (Figure 1). Due to the acquired information, the following day, Istituto Zooprofilattico Sperimentale della Puglia e della Basilicata (IZSPB) staff investigated the cattle close to the patient’s farm for ticks and related Spotted Fever Group (SFG) rickettsiae. The delivered tick specimen from the patient and 15 hard ticks collected from five grazing cattle during the IZSPB investigation were morphologically identified according to Manilla morphological keys [13] and subjected to total DNA extraction using the DNeasy blood and tissue extraction kit (Qiagen, Milan, Italy). The attempts at molecular amplification by conventional PCR of the 17 kDa outer membrane protein gene (*17kDa*) and the citrate synthase-encoding gene (*gltA*) from the specimen as described by Webb and Roux [14,15] were successful. The gathered nucleotide sequences (Acc. numbers ON502850, ON502851) were compared with those present in GenBank and the identification of the rickettsia species was confirmed. All of the 16 examined ticks were identified as engorged female of *Hyalomma marginatum marginatum* (*H. m. m.*). Sequencing of the PCR products showed 100% identity with *Rickettsia* (*R.*) *aeschlimannii*. Due to the circumstances of the events two years earlier, it was impossible to determine the presence of *R. aeschlimannii* by molecular tests, rickettsia isolation or immunohistochemistry in the patient’s lesions. Unfortunately, despite our request for a serological test, the patient found the blood draw inappropriate. The cattle farmer, in fact, considered the molecular detection of the pathogen in the preserved tick sufficient to solve his diagnostic doubt.

## 3. Discussion and Conclusions

**Figure 2:**
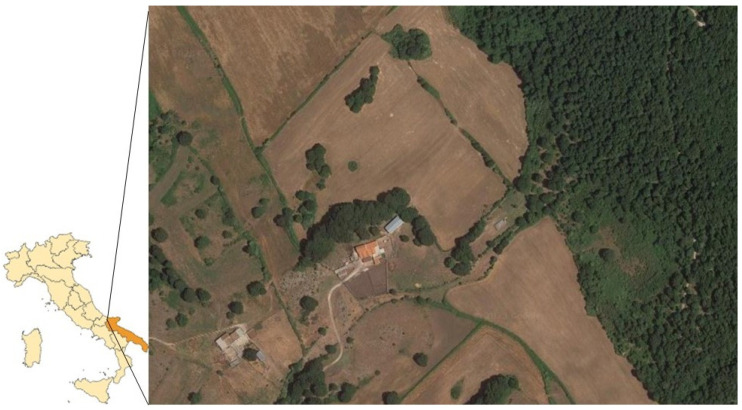
Location of farm: site with details of cattle breeding areas with pastures and bush.

In the Mediterranean area, *H. marginatum*, along with *Ixodes ricinus*, *Dermacentor marginatus and Rhipicephalus sanguineous*, is regarded as one of the most common tick species of medical interest. In Italy, the genus *Hyalomma* Koch, 1844 includes the species *H. aegyptium, H. lusitanicum, H. detritum* (formerly *H. d. detritum* and *H. d. scupense* subspecies), *H. excavatum* and *H. marginatum* (formerly *H. m. marginatum* and *H. m. rufipes* subspecies) [13,16]. The ticks of the *H. marginatum* complex are characterized by a high ecological plasticity and they can be found in very different environments, including arid or humid pastures, in low, medium and high mountains or in the Mediterranean maquis. In Italy, adult and immature specimens of *H. m. marginatum* were historically reported in continental and insular regions, including the Apulia region [17,18,19]. *H. marginatum* is an exophilic and ditropic tick with a cycle of two hosts where only the larvae and adults need to find a host. Adults usually feed on large mammals, mainly artiodactyls and perissodactyls, while the immature forms are commonly ectoparasites of lagomorphs, rodents and of a wide number of birds [13]. The ability to molt on birds decreases the duration of the tick life cycle and also facilitates its environmental dispersion [20]. In all the development stages of the ticks, the specimens exhibit a hunter strategy compared to the questing strategy in most tick genera. In fact, they actively run towards a suitable host and the adults show an aggressive behavior when they parasitize mainly domestic and wild ruminants and h-mans. *H. marginatum* is also a well-known vector and reservoir for various zoonotic and emerging pathogens, including Crimean–Congo hemorrhagic fever (CCHF) virus and *R. aeschlimannii*; both these pathogens can persist in the arthropod for their entire lifespan, and they can be also transmitted vertically to the next generation. The Mediterranean basin’s warmer parts, in fact, house an autochthonous population of H marginatum but the evidence of a trend towards a warmer climate in Europe and the probability of introduction of immature specimens by birds has increased the recognition of this tick species as a possible invasive species for the countries of northern Europe [21] with the associated risk of introducing and maintaining both cited pathogens. Furthermore, *H. marginatum* is considered to be the main reservoir of *R. aeschlimannii* [22,23,24,25]. On cattle, adult ticks typically appear in congregations, feeding on udders, the scrotum, inguinal area and perineum. Co-feeding is one of the primary methods of pathogen transmission in the tick clusters; however, potential pathogens can also be transmitted by *H. m. m* in a transstadial way. *H. marginatum* can transmit *R. aeschlimannii* to future generations through transovarian transmission, promoting the ability of this tick species to maintain and colonize new territories, hosts and environments. Birds, cattle, small ruminants, camels and rodents, although asymptomatic, could be the vertebrate reservoirs of the pathogen in infected areas. The ability of *R. aeschlimannii* to infect other extremely prolific tick and mammal species has been also reported. In Algeria, in fact, the DNA of *R. aeschlimannii* has been recently amplified in specimens of *Rhipicephalus bursa* (Anatolian brown tick) collected from cattle and in Tunisia the bacterium was isolated from wild rodents [26]. In our case, the breeding of Podolican cattle, raised in the wild, was located about 450 m above sea level, in a wooded area with deciduous trees and pastures with undergrowth populated by ferns, brambles and wild roses. Cattle farming is managed internally and the animals are free to move in 300 ha of pasture (Figure 2). The breeder often accompanies the animals on the pasture wearing simple rubber boots. When questioned, the cattle farmer reported a seasonal increase in tick infestations from May to September, especially in the days following the rain and he usually used tick repellent products to protect his animals. The photo-story archived in his smartphone was chronologically detailed and it showed the progression of the cutaneous lesions and the remission of the symptoms after using doxycycline. The patient reported that he had previously suffered from tick bites without development of clinical symptoms; in the cited episodes, the observed tick specimens were different in size and color than the *R. aeschlimannii*-infected tick specimen. After the *H. marginatum* bite, his foot quickly became so swollen that he could not wear shoes and the pain was so intense that it could not walk. The patient’s awe increased due to the onset of the fever and to the worsening of the skin lesion caused by the tick bite. He did not report the appearance of spots or a maculopapular rash. During this time, the clinician contacted by telephone after assessing the symptoms and history suspected a tick bite infection, such as Lyme disease or rickettsiosis.

Due to the lack of available serological tests in the medical laboratory and the severe restrictions associated with the SARS-CoV-2 infection, therapy was prescribed for suspected rickettsiosis. The *tache noir* fell and the wound improved gradually after receiving antibiotic treatment with doxycycline. The patient’s cousin took care of the cattle during his convalescence and did not report any health problems. Although the general condition of the patient improved quickly and the swelling and pain of the foot disappeared after about 7 days, at the site of the tick bite, the healing of the skin was very slow. About 600 days after the parasite removal, a skin area with abnormal pigmentation was still visible. The cattle examined were in good health and showed no signs of infectious disease. The IZSPB database consultation confirmed that, in addition to *H. marginatum*, several species of ticks have been previously collected in the investigated area, such as *I. ricinus*, *D. marginatus*, *Rh. turanicus, Rh. sanguineus, Rh. bursa* and *Haemaphysalis inermis*; furthermore, zoonotic tick-borne pathogens like *Borrelia garinii* and *B. afzelii* [27], *R. helvetica*, *R. raoultii*, *R. slovaca*, *R. felis* [28] and *R. monacensis* had also been identified in questing ticks collected in the same area. The detection of these pathogens in competent vectors suggests that the number of human infections related to tick-borne diseases could be higher than the scarcely reported cases in the medical literature from Southern Italy, including the Gargano area. This is supplemented with the fact that the diagnosis of *R. aeschlimannii* infection may be extremely challenging; in fact, in addition to the MSF manifestations, characterized by eschar, rash, edema and partial necrosis surrounding the tick bite site, episodes may present with nonspecific clinical symptoms (fever, headache, myalgia, vomiting, hepatitis, meningitis, etc.), without cutaneous signs, such as observed in the case of an infection acquired in Southern Italy [3], or may be totally asymptomatic [29]. *R. aeschlimannii* could be the cause of necrosis, erythema, myalgia, edema, and partial necrosis surrounding the tick bite site with clinical manifestations analogous to those described in several infections with SFGR. The clinical signs illustrated in our study are very similar to a report case of an *R. aeschlimannii* infection in a woman in China [30]. In the clinical case recorded by Yang, the patient’s symptoms, the location of the tick bite and the medical therapy are analogous to our case. The authors also performed research and subsequent detection of *R. aeschlimannii* in ticks around the patient’s home and they associated the human infection with the *R. aeschlimannii* detected in vectors. On the basis of the above, a diagnosis of rickettsiosis is still a challenge, especially when it is solely based on clinical manifestations. Since the development of the Weil-Felix test, serology has been the primary method for diagnosing rickettsial infections. Increased sensitivity and specificity have been achieved by advances in serological analysis, but several critical aspects still remain unsolved. Among these limits is the correct identification of species. *R. rickettsii* or *R. conorii* antigens are the only ones used in many commercial assays that use indirect immunofluorescence assays (IFAs). Discarding Rickettsia groups but not species can also be achieved by using an enzyme immunosorbent assay (ELISA) for IgM or IgG for SFG and Typhus Group rickettsial infections. An alternative technique that has been shown to be effective for the differentiation of antibodies against different Rickettsia species is the micro-IFA (MIF). The assay was able to discriminate specific antibodies for *R. helvetica*, *R. raoultii* and *R. slovaca* in samples of canine serum [31] and *R. aeschlimannii*, *R. conorii*, *R. africae*, *R. slovaca*, *R. helvetica* and *R. massiliae* in human serum samples [6]. On the other hand, the rickettsia MIF is expensive, requires technical expertise, and is limited to reference laboratories. We therefore believe that previous exposures to the patient’s tick bites in addition to the lack of species-specific serological diagnostic kits and the time elapsed since the onset of infection, were all conditions certainly unfavorable for the purpose of diagnosis of the cattle breeder. Consequently, even if the patient had consented to blood sampling for serological screening in our case, the results would have been difficult to interpret after almost two years of infection. Nucleic acid amplification and immunohistochemistry testing are the current methods for detecting Rickettsia species in clinical samples. Blood samples, serum, tissues, tampons from ulcers and scabs [32] and of course also blood-sucking arthropods are currently used to yield a direct diagnosis of rickettsial infection. Molecular diagnostics and amplification of nucleic acids have been shown to be techniques with high sensitivity and specificity and are widely applied in research as well as in diagnostics. In our case, the study of sequences yielded from the *17kDa* and *gltA* genes allowed us to characterize the species detected as *R. aeschlimannii*. In addition, entomological findings show that the investigated area houses several specimens of *H. m. m.* that are potentially dangerous for human health, highlighting that the percentage of ticks collected and found to be infected was 100%. There is still a lack of information regarding the role of mammals or birds as potential reservoirs of zoonotic rickettsiae. Despite the identification of *R. helvetica* DNA in deer blood samples [33], the presence of pathogenic rickettsiae, including *R. aeschlimannii*, in domestic and wild ruminants is still largely uninvestigated. In conclusion, due to the high prevalence of *R. aeschlimannii* in the examined ticks and technical difficulties of making a retrospective serological diagnosis of infection among the SFG rickettsiae, we suppose that there is a fairly plausible correlation between the removed tick and the clinical manifestation of the patient. The history and the clinical signs are highly presumptive of tick-borne rickettsiosis. Although the presence of *R. aeschlimannii* has been ascertained only in ticks, further investigations in vertebrate hosts should be recommended to identify the vertebrate reservoirs of *R. aeschlimannii* in the Mediterranean region. In sites where ticks are present, the differential diagnosis between the various cutaneous clinical findings has certainly been a medical challenge. Interestingly, there is the evidence that both COVID-19 (SARS-CoV-2) and rickettsial infection can have cutaneous involvement with eschar and necrotic lesions; in fact, both share a similar mechanism of infecting endothelial cells, resulting in vasculopathy lesions [34]. However, a careful anamnesis may promptly reveal a suspected exposure to arthropods, particularly in subjects involved in animal management, such as farmers. In Italy, the restrictions accompanying to the viral pandemic have made the diagnosis of neglected diseases, including tick-borne diseases, even more difficult; in addition, the progression of the treatment and the follow-up were often communicated by telephone from patients to the clinicians. In the context of restrictions linked to the COVID-19 pandemic, the doctor–patient relationship has also benefited from a technological contribution linked to new communication technologies, particularly in rural areas far from medical facilities. In our study, the preservation of the arthropod responsible for the bite by the patient allowed us to carry out, about 2 years later, a retrospective molecular identification of the pathogen. 

## Data Availability

GenBank database Acc. numbers ON502850 and ON502851.

## References

[1] Germanakis A., Chochlakis D., Angelakis E., Tselentis Y., Psaroulaki A. (2013). *Rickettsia aeschlimannii* infection in a man, Greece. Emerg. Infect. Dis..

[2] Socolovschi C., Parola P., Raoult D., Magill A.J., Hill D.R., Solomon T., Ryan E.T. (2013). Tick-borne Spotted Fever Rickettsioses. Hunter’s Tropical Medicine and Emerging Infectious Disease.

[3] Tosoni A., Mirijello A., Ciervo A., Mancini F., Rezza G., Damiano F., Cauda R., Gasbarrini A., Addolorato G., Internal Medicine Sepsis Study Group (2016). Human *Rickettsia aeschlimannii* infection: First case with acute hepatitis and review of the literature. Eur. Rev. Med. Pharmacol. Sci..

[4] Znazen A., Rolain J., Hammami N., Hammami A., Ben Jemaa M., Raoult D. (2006). *Rickettsia felis* Infection, Tunisia. Emerg. Infect. Dis..

[5] Beati L., Meskini M., Thiers B., Raoult D. (1997). *Rickettsia aeschlimannii* sp. nov., a new spotted fever group rickettsia associated with *Hyalomma marginatum* ticks. Int. J. Syst. Bacteriol..

[6] Raoult D., Fournier P.E., Abboud P., Caron F. (2002). First documented human *Rickettsia aeschlimannii* infection. Emerg. Infect. Dis..

[7] Oteo J.A., Portillo A. (2012). Tick-borne rickettsioses in Europe. Ticks Tick-Borne Dis..

[8] Igolkina Y., Rar V., Krasnova E., Filimonova E., Tikunov A., Epikhina T., Tikunova T. (2022). Occurrence and clinical manifestations of tick-borne rickettsioses in Western Siberia: First Russian cases of *Rickettsia aeschlimannii* and *Rickettsia slovaca* infections. Ticks Tick-Borne Dis..

[9] Guccione C., Colomba C., Tolomeo M., Trizzino M., Iaria C., Cascio A. (2021). Rickettsiales in Italy. Pathogens.

[10] Blanda V., Torina A., La Russa F., D’Agostino R., Randazzo K., Scimeca S., Giudice E., Caracappa S., Cascio A., de la Fuente J. (2017). A retrospective study of the characterization of Rickettsia species in ticks collected from humans. Ticks Tick-Borne Dis..

[11] Chisu V., Zobba R., Foxi C., Pisu D., Masala G., Alberti A. (2016). Molecular detection and groEL typing of *Rickettsia aeschlimannii* in Sardinian ticks. Parasitol. Res..

[12] Battisti E., Urach K., Hodžić A., Fusani L., Hufnagl P., Felsberger G., Ferroglio E., Duscher G.G. (2020). Zoonotic Pathogens in Ticks from Migratory Birds, Italy. Emerg. Infect. Dis..

[13] Manilla G. (1998). Acari Ixodida, Fauna d’Italia.

[14] Webb L., Mitchell C., Malloy D.C., Dasch G.A., Azad A.F. (1990). Detection of murine typhus infection in fleas by using the polymerase chain reaction. J. Clin. Microbiol..

[15] Roux V., Fournier P.E., Raoult D. (1996). Differentiation of spotted fever group rickettsiae by sequencing and analysis of restriction fragment length polymorphism of PCR- amplified DNA of the gene encoding the protein rOmpA. J. Clin. Microbiol..

[16] Apanaskevich A.D., Horak I.G. (2008). The genus *Hyalomma* Koch, 1844: V. re-evaluation of the taxonomic rank of taxa comprising the *H. (Euhyalomma) marginatum* koch complex of species (Acari: Ixodidae) with redescription of all parasitic stages and notes on biology. Int. J. Acarol..

[17] Sobrero L., Manilla G. (1988). Aggiornamenti Sulle Zecche d’Italia.

[18] Toma L., Mancini F., Di Luca M., Cecere J.G., Bianchi R., Khoury C., Quarchioni E., Manzia F., Rezza G., Ciervo A. (2014). Detection of microbial agents in ticks collected from migratory birds in central Italy. Vector Borne Zoonotic Dis..

[19] Mancuso E., Di Domenico M., Di Gialleonardo L., Menegon M., Toma L., Di Luca M., Casale F., Di Donato G., D’Onofrio L., De Rosa A. (2023). Tick Species Diversity and Molecular Identification of Spotted Fever Group Rickettsiae Collected from Migratory Birds Arriving from Africa. Microorganisms.

[20] Hoffman T., Carra L.G., Öhagen P., Fransson T., Barboutis C., Piacentini D., Figuerola J., Kiat Y., Onrubia A., Jaenson T.G.T. (2021). Association between guilds of birds in the African-Western Palaearctic region and the tick species *Hyalomma rufipes*, one of the main vectors of Crimean-Congo hemorrhagic fever virus. One Health.

[21] Estrada-Peña A., D’Amico G., Fernández-Ruiz N. (2021). Modelling the potential spread of *Hyalomma marginatum* ticks in Europe by migratory birds. Int. J. Parasitol..

[22] Parola P., Paddock C.D., Raoult D. (2005). Tick-borne rickettsioses around the world: Emerging diseases challenging old concepts. Clin. Microbiol. Rev..

[23] Keysary A., Eremeeva M.E., Leitner M., Din A.B., Wikswo M.E., Mumcuoglu K.Y., Inbar M., Wallach A.D., Shanas U., King R. (2011). Spotted fever group rickettsiae in ticks collected from wild animals in Israel. Am. J. Trop. Med. Hyg..

[24] Abdel-Shafy S., Allam N.A., Mediannikov O., Parola P., Raoult D. (2012). Molecular detection of spotted fever group rickettsiae associated with ixodid ticks in Egypt. Vector Borne Zoonotic Dis..

[25] Gargili A., Palomar A.M., Midilli K., Portillo A., Kar S., Oteo J.A. (2012). Rickettsia species in ticks removed from humans in Istanbul, Turkey. Vector Borne Zoonotic Dis..

[26] Abdelbaset A.E., Kwak M.L., Nonaka N., Nakao R. (2023). Human-biting ticks and zoonotic tick-borne pathogens in North Africa: Diversity, distribution, and trans-Mediterranean public health challenges. One Health.

[27] Raele D.A., Pugliese N., Galante D., Cafiero M.A. (2019). Loop-mediated isothermal amplification in the field: Reality beyond the potentiality. Turk. J. Vet. Anim. Sci..

[28] Raele D.A., Galante D., Pugliese N., Salandra G., Cafiero M.A. (2018). Spotted fever group rickettsiae associated with ixodid ticks in wild environment in Southern Italy. Microbiologyopen.

[29] Koczwarska J., Pawełczyk A., Dunaj-Małyszko J., Polaczyk J., Welc-Falęciak R. (2023). Rickettsia species in *Dermacentor reticulatus* ticks feeding on human skin and clinical manifesta-tions of tick-borne infections after tick bite. Sci. Rep..

[30] Yang M., Jia Y., Dong Z., Zhang Y., Xie S., Liu Q., Wang Y. (2022). *Rickettsia aeschlimannii* Infection in a Woman from Xingjiang, Northwestern China. Vector Borne Zoonotic Dis..

[31] Wächter M., Wölfel S., Pfeffer M., Dobler G., Kohn B., Moritz A., Pachnicke S., Silaghi C. (2015). Serological differentiation of antibodies against *Rickettsia helvetica*, *R. raoultii*, *R. slovaca*, *R. monacensis* and *R. felis* in dogs from Germany by a micro-immunofluorescent antibody test. Parasit. Vectors.

[32] Stewart A.G., Stewart A.G.A. (2021). An Update on the Laboratory Diagnosis of *Rickettsia* spp. Infection. Pathogens.

[33] Grassi L., Drigo M., Zelená H., Pasotto D., Cassini R., Mondin A., Franzo G., Tucciarone C.M., Ossola M., Vidorin E. (2023). Wild ungulates as sentinels of flaviviruses and tick-borne zoonotic pathogen circulation: An Italian perspective. BMC Vet. Res..

[34] Mittal A., Elias M.L., Schwartz R.A., Kapila R. (2021). Recognition and treatment of devastating vasculopathic systemic disorders: Coronavirus disease 2019 and rickettsioses. Dermatol. Ther..

